# Nursing Pillows in the Sleep Environment and Sudden Unexpected Infant Deaths — Georgia, January 2013–December 2022

**DOI:** 10.15585/mmwr.mm7419a2

**Published:** 2025-05-29

**Authors:** Bridget K. Hamilton, Terri Miller, Robin Dawson

**Affiliations:** ^1^University of South Carolina College of Nursing, Columbia, South Carolina; ^2^Jiann-Ping Hsu College of Public Health, Georgia Southern University, Statesboro, Georgia.

**Summary**

**What is already known about this topic?**

Sudden unexpected infant deaths (SUIDs) often occur in spaces where infants sleep. In 2008, the Consumer Product Safety Commission exempted pillows used to support infants during feeding (nursing pillows) from a ban on infant pillows.

**What is added by this report?**

Analysis of Child Death Review data found that among 1,685 SUIDs in Georgia during 2013–2022, a nursing pillow was in the infant’s sleep space in 84 (5%) cases. Eighty percent of these deaths were in infants aged <4 months, 56% occurred in an adult bed, and all but one involved bed sharing.

**What are the implications for public health practice?**

Nursing pillows are not intended for use in sleep spaces for infants. Warnings on new product labels and continued education and outreach about safe infant sleep could help reduce SUIDs.

## Abstract

A sudden unexpected infant death (SUID) is defined as the sudden and unexpected death of an infant (a child aged <1 year) whose cause of death was not obvious before investigation. Pillows used to support infants during feeding, often referred to as nursing pillows, have been identified as a potential hazard in sleep spaces for infants. Georgia county-level Child Death Review (CDR) data from the Pediatric National Fatality Review Case Reporting System were analyzed to ascertain whether nursing pillows were found in the sleep space of infants who died of SUID. Among 1,685 SUID cases in Georgia during 2013–2022, a nursing pillow was found in the sleep space of 84 (5%) infants. Among these, 86% of infants who died with a nursing pillow present were aged <4 months, 40% were aged <2 months, and 55% were Black or African American. A total of 56% of the deaths occurred in an adult bed, and all but one (99%) occurred in association with bed sharing. Among the 84 deaths, the nursing pillow was found under the infant in 58 (69.1%) cases, next to the infant in 14 (16.7%) cases, on top of the infant in two (2.4%) cases, and tangled around the infant in one (1.2%) case. This analysis indicates that nursing pillows are being used in ways other than their intended use as an aid in feeding. Since April 2025, newly manufactured nursing pillows must have labels indicating the potential risk associated with using them in infants’ sleep spaces; however, many nursing pillows in use or still on the market lack such labeling. Warning consumers of risks associated with using nursing pillows in infant sleep environments, in addition to continued education and outreach about safe infant sleep, could help reduce SUIDs.

## Introduction

Each year, approximately 3,700 infants in the United States die from sudden unexpected infant death (SUID) (*1*,*2*), defined as the sudden and unexpected death of a child aged <1 year (an infant) for whom the cause of death was not obvious before an investigation (*1*,*2*). Causes of death attributed to SUID include sudden infant death syndrome (SIDS) and other deaths of unknown cause, as well as accidental suffocation or strangulation in the sleeping environment (*2*). Sleep practices to which accidental suffocation or strangulation have been attributed include not placing infants on their backs, or supine, to sleep (i.e., prone placement), including soft bedding (e.g., blankets and stuffed toys) in the infant’s sleep space, and not placing the infant in a separate designated sleep space (i.e., a crib or bassinet) (*2*).

Infant cushions and pillows have also been identified as hazards in the sleep environment. In 1992, the Consumer Product Safety Commission (CPSC) issued an infant pillow ban, barring promotion of “infant cushions,” “infant pillows,” and similar articles (e.g., pillows loosely filled with granular material, easily flattened, or capable of conforming to the body or face of an infant) intended for use by children aged <1 year (*3*).

In 2008, CPSC approved an exemption to the infant pillow ban for pillows used for breastfeeding support (nursing pillows) using CPSC data from January 1992–May 2008 because these products are intended to perform a function that is different from that of infant cushions (*3*). Nursing pillows are firm, tubular crescent-shaped, U-shaped, or round cushions that fit on or around the caregiver’s body and are intended to aid in infant feeding by providing ergonomic support to the caregiver and raising the infant’s head while feeding (*3*). They are commonly filled with synthetic batting or foam, cotton, wool, or dried grains (*3*). The exemption also permitted voluntary manufacturer labeling of nursing pillows regarding their intended use.

An analysis of 2004–2015 U.S. Pediatric National Fatality Review Case Reporting System (NFR-CRS) data (*4*) found that during this period, a nursing pillow was present in association with 141 sleep-related deaths nationwide (*5*). Researchers classified infant deaths with nursing pillows as infrequent because they found so few cases nationwide; however, they did not disclose how cases were identified in their national sample or whether specific variables were used to screen for cases, resulting in challenges associated with validating those findings.

In Georgia, the number of infant deaths attributed to accidental suffocation or strangulation has increased since 2011.* This finding highlights the need to assess how recurrently objects that are known risks to infants in sleep environments, such as nursing pillows, are identified as a factor in sleep-related infant deaths. NFR-CRS data were analyzed to characterize the presence of nursing pillows in the infant sleep space using SUID investigation reports in Georgia during 2013–2022.

## Methods

### Data Source

NFR-CRS data from Georgia for January 2013–December 2022 were derived from the National Center for Fatality Review and Prevention’s (*4*) Pediatric National Fatality Review Case Reporting System.^†^ The National Center for Fatality Review and Prevention is a national resource and data center for Fetal and Infant Mortality Review and Child Death Review (CDR). CDR is a collaborative process involving partners from multiple disciplines to review selected cases within their jurisdiction and document the circumstances leading to the death of a child in an effort to identify risk factors that might guide development and implementation of strategies to prevent future deaths.^§^ NFR-CRS data are collected from county-level CDRs who submit data to the national center. Not all deaths are reviewed; however, the goal is to review a representative sample of cases that occurred within the jurisdiction. Between 2013 and 2022, a total of 1,685 SUID cases were reviewed by local CDR teams throughout Georgia.

### Identification of Factors Associated with SUIDs

SUID case reports include a variable within the data set that indicates 1) whether the death was sleep related and 2) whether the cause of death was asphyxia or undetermined. Asphyxia, sleep-related, and undetermined SUID and SIDS cases that underwent CDR are included in this analysis. All cases included in this analysis were linked to verifiable death certificates to eliminate duplicate cases.

The NFR-CRS includes coded variables and narratives that include details about each case. Selected coded variables are those identified by the CDR team as possible substantial factors in a sleep-related death. Cases were first identified as those that included a nursing pillow selected as a possible factor in the sleep environment. In some instances, the narrative about the death might contradict the variable selected as a contributing factor. Therefore, to determine whether a death likely resulted from a nursing pillow in the infant’s sleep environment, two researchers reviewed each case and used information in the narratives to screen out cases (i.e., those for which the cause of death was clearly attributable to something other than the presence of a nursing pillow in the sleep environment). For example, SUID or SIDS cases that were suspected homicides were excluded, even if a nursing pillow was present in the infant’s sleep space. Interrater reliability was 100%. Descriptive statistics were generated using Microsoft Excel. This project was reviewed by the Georgia Department of Public Health Institutional Review Board and was deemed non–human-subjects research.

## Results

Among 1,685 SUID cases that occurred during 2013–2022 and were reviewed, a nursing pillow was in the infant’s sleep space in 90 (5.3%) cases. Six cases were excluded from additional analysis, including two attributable to potential homicide and two to medical conditions (one case in an infant with extreme prematurity and one in an infant with a condition not stated but implied to be an unspecified respiratory virus). Two additional cases were excluded, both of which involved an infant being placed inside a crib or portable playpen while buckled into a car seat or swing, with a nursing pillow in the crib or playpen but not on or near the infant. The remaining 84 (5%) cases met the inclusion criteria of being an SUID with a nursing pillow in the infant sleep space.

The number of SUIDs involving nursing pillows in the infant sleep space and reviewed by CDR teams increased from three in 2013 to 14 in 2022 (Figure).^¶^ Among the 84 total SUIDs associated with nursing pillows during 2013–2022, Black or African American and White** infants accounted for approximately one half (46, 54.8%) and one third (27, 32.1%) of cases, respectively (Table). Fifty (59.5%) deaths occurred in boys, and 34 (40.5%) in girls. All SUIDs occurred in infants aged <9 months; 86% of these deaths occurred in infants aged <4 months, and 40% in infants aged <2 months. Other identified factors associated with SUIDs involving nursing pillows included sleeping in an adult bed (56.0%) and formula feeding (17.9%). Information about behaviors such as propping up bottles to feed infants, specific feeding methods, or formula feeding and breastfeeding was not included in this dataset. All cases but one (99%) were associated with bed sharing. The location of the pillow relative to the infant varied and included being found under the infant (69.0% of cases), next to the infant (16.7%), on top of the infant (2.4%), or tangled around the infant (1.2%). In 10.7% of cases, information on placement of the nursing pillow relative to the infant was not available.

**FIGURE:**
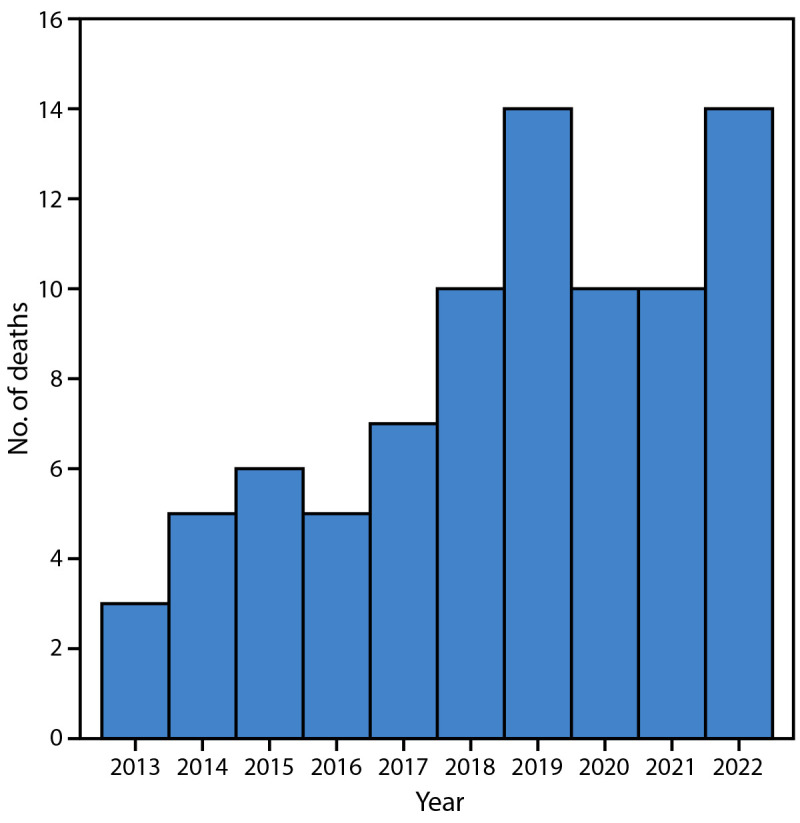
Sudden unexpected infant deaths* with a nursing pillow in the sleep environment (N = 84) — Georgia, 2013–2022^†^ * Sudden unexpected death of an infant aged <1 year whose cause of death was not obvious before investigation. ^†^ Data are from an analysis of Georgia county-level Child Death Review data from the Pediatric National Fatality Review Case Reporting System, including 1,685 (86.5%) of 1,948 sudden unexpected infant deaths.

**TABLE:** Sudden unexpected infant deaths with a nursing pillow in the sleep environment, by demographic characteristics and risk factors — Georgia, 2013–2022

Characteristic	No. (%)* (N = 84)
**Race and ethnicity^†^**	
Black or African American	46 (54.8)
Hispanic or Latino	3 (3.6)
White	27 (32.1)
Multiracial or other	8 (9.5)
**Infant’s age at death, mos**	
<1	6 (7.1)
1	28 (33.3)
2	20 (23.8)
3	13 (15.5)
4	5 (6.0)
5	3 (3.6)
6	2 (2.4)
7	3 (3.6)
8	4 (4.8)
**Sex**	
Female	34 (40.5)
Male	50 (59.5)
**Sleep space**	
Adult bed	47 (56.0)
Crib	19 (22.6)
Other	17 (20.2)
Unknown	1 (1.2)
**Other risk factors^§^**	
Bed sharing between infant and other person	83 (98.8)
Formula-fed infant	15 (17.9)
Toys in infant’s sleep area	11 (13.1)
Smoking^¶^	9 (10.7)
Alcohol^¶^	7 (8.3)
Marijuana or amphetamines^¶^	2 (2.4)
**Position of nursing pillow**	
Under infant	58 (69.0)
Next to infant	14 (16.7)
On top of infant	2 (2.4)
Tangled around infant	1 (1.2)
Unknown	2 (2.4)
Missing	7 (8.3)

* Data are from an analysis of Georgia county-level Child Death Review data from the Pediatric National Fatality Review Case Reporting System, including 1,685 (86.5%) of 1,948 sudden unexplained infant deaths.

^†^ Hispanic or Latino (Hispanic) was categorized as a race rather than an ethnicity. Therefore, the Hispanic ethnicity of infants categorized as Black or African American, White, multiracial, or another race is not known.

^§^ Risk factors were not mutually exclusive.

^¶^ The primary person supervising the infant at the moment of the incident or when the death investigation was being conducted was actively smoking or impaired by alcohol, marijuana, or methamphetamines.

## Discussion

Despite warnings from CPSC, nursing pillow manufacturers, and the American Academy of Pediatrics (AAP) about the dangers posed by nursing pillows in sleep spaces for infants (*3*), the number of SUID cases with a nursing pillow present as a possible factor in a sleep-related infant death increased in Georgia from 2013 to 2022. The presence of any soft object, including a nursing pillow, in an infant sleep space is a risk factor for SUID (*2*). A mandatory federal safety standard proposed by CPSC in September 2024 and implemented in April 2025 includes new warning label requirements for nursing pillows.^††^ Although all nursing pillows now have a label, current marketing strategies by certain nursing pillow manufacturers and retailers do not stipulate that the pillow is intended only for infant feeding and suggest in promotional images or directly state in other advertisements that the pillow can be used for infant “lounging.” This can be confusing for consumers, some of whom might assume that nursing pillows are intended to be used to support sleeping infants.

In 17.9% of SUIDs with a nursing pillow as a possible factor in the death, the infant was confirmed to be formula-fed; however, information about whether a bottle was found in the infant sleep space was not available. Because breastfeeding has been shown to be protective against SUID (*6*), community groups have found that distributing nursing pillows can encourage breastfeeding (*7*). However, products used to facilitate breastfeeding should not pose unintended risks to the infant. This analysis highlights the importance of keeping soft objects out of the infant sleep space and of educating caregivers about the intended use of these pillows, and the potential risks associated with using them in infants’ sleep environments, when distributing them in the community to promote breastfeeding.

These data provide additional evidence that nursing pillows should not be placed in infants’ sleep spaces and should not be used for shared surfaces. The majority of cases in this study occurred in the adult bed, providing additional data indicating that adult beds are not a safe surface for infants, even if a nursing pillow is used. Community advocates, health care providers, and nurses could play a critical role in educating families about the ABC’s (alone, back, crib) of safe infant sleep before hospital discharge, at the 1- or 2-week pediatric primary care follow-up, and at the 2-month follow-up visit. These are all convenient and ideal times for reinforcing education about safe sleep practices and proper use of the nursing pillows.

### Limitations

The findings in this report are subject to at least five limitations. First, CDR data are limited to the information collected by CDR teams at the county level and likely underrepresent the number of deaths. Second, although CDRs are mandated by Georgia law, not all deaths among children are reviewed quickly, accurately, or systematically, which limits the ability to infer causal relationships.^§§^ Therefore, the cases reviewed for this study likely do not reflect the total number of SUIDs that occurred in Georgia during 2013–2022. Third, the data are limited by the information collected during the death investigation; for the cases in this study, the exact role of the nursing pillow in the death was either unknown or undocumented. Fourth, the postmortem medical diagnoses for SIDS and SUID are similar, and it is possible that certain deaths included in this analysis should have been excluded because they were attributable to a specific cause of death, such as a respiratory virus or medical complication. However, because the data were missing, these deaths could not be screened out; steps were taken to reduce this to the extent possible (*8*). Finally, the demographic data captured in this data set are limited by incomplete data collection and misclassification. For example, although major metropolitan areas within Georgia are home to large numbers of Asian populations, no deaths among Asian infants were captured. Details about cultural practices and behaviors that might affect the sleep of infants are not systematically captured by this dataset, which inherently limits identification of subpopulations that might disproportionately use nursing pillows in infant sleep spaces.

### Implications for Public Health Practice

This study highlights how use of state and local child death review data via NCFRP can guide understanding of a widespread practice with public health implications. The national Safe to Sleep campaign (originally the Back to Sleep Campaign in 1994),^¶¶^ has played a substantial role in promoting strategies to reduce sleep-related deaths among infants. Given the increase in sleep-related infant deaths nationwide (*9*) and within Georgia, public health programs to continue this work are essential (*2*). Some products meant to ease infant care and support parents might contribute to SUID risk if they are not appropriately labeled and used as intended. Until April 2025, nursing pillows lacked labeling describing the potential hazards of using the products in infant sleep spaces, which could contribute to the number of deaths identified in this analysis. The new CPSC safety standard for labeling was implemented in April, with warnings that infants have died while using nursing pillows for sleep or lounging, infants can suffocate within minutes, and the product should only be used to feed infants who remain awake. These new labeling requirements might help prevent infant deaths associated with using nursing pillows being used in ways other than their intended use as a feeding aid.

However, even with new labeling requirements, nursing pillows could continue to pose a risk in sleep spaces for infants. Public health interventions such as messaging for parents, caregivers, and health care providers about the dangers of using nursing pillows in an unintended way could prevent deaths among infants. National evidence-based guidelines, in coordination with AAP guidelines, could help provide consistent messaging, educational materials, and warnings among jurisdictions, while guiding research and interventions to reduce infant deaths. 

## Footnotes

*
Georgia Department of Public Health. Online Analytical Statistical Information System (OASIS)

†
National Center for Fatality Review and Prevention

§
National Center for Child Death Review. A Program Manual for Child Death Review

¶ The COVID-19 pandemic affected the number of cases reviewed during 2020 and 2021.

** Hispanic or Latino (Hispanic) was categorized as a race rather than an ethnicity. Therefore, the Hispanic ethnicity of infants categorized as Black or African American, White, multiracial, or another race is not known.

†† CPSC Approves New Federal Safety Standard for Nursing Pillows to Prevent Infant Deaths and Serious Injuries - September 2024; Federal Register / Vol. 89, No. 207 / Oct 25, 2024. Consumer Product Safety Commission. Safety Standard for Nursing Pillows

§§ Not all deaths in Georgia are reviewed, and certain counties do not comply with completing CDRs.

¶¶ National Institutes of Health. Safe Sleep for Your Baby: Reduce the Risk of Sudden Infant Death Syndrome (SIDS) and Other Sleep-Related Infant Deaths; National Institutes of Health. Safe to Sleep Campaign History
